# Perceptions of the usefulness of Choosing Wisely among general practitioners in Norway: a nationwide survey

**DOI:** 10.1186/s12875-025-02928-5

**Published:** 2025-08-02

**Authors:** Jørgen Breivold, Karin Isaksson Rø, Stein Nilsen, Stefán Hjörleifsson

**Affiliations:** 1 Department of Global Public Health and Primary Care, University of Bergen, PO Box 7804, Bergen, N-5020 Norway; 2 The Norwegian Medical Association, Institute for Studies of the Medical Profession, Oslo, Norway; 3 The Norwegian Labour and Welfare Administration, Bergen, Norway

**Keywords:** General practice, Gatekeeping, Decision making, Low-value care, Medical overuse, Survey

## Abstract

**Background:**

Choosing Wisely is an international initiative to curb medical overuse. The Norwegian College of General Practice has published recommendations to avoid commonly used services for safer healthcare. This study investigated Norwegian GPs’ perceptions of Choosing Wisely.

**Methods:**

Cross-sectional online survey of Norwegian GPs in 2021. We report GPs’ perceptions of Choosing Wisely recommendations as proportions, using multiple ordinal regression to assess factors influencing the campaign’s perceived usefulness.

**Results:**

Responses from 900 GPs, with a response rate of 18% were included. 81% were aware of Choosing Wisely, and of these 82% found the campaign somewhat or very useful in reducing overdiagnosis or overtreatment. This correlated with lower levels of work-related stress (adjusted odds ratio (AOR) 0.61, 95% confidence interval (CI) 0.45–0.83) and more frequently reaching an agreement with patients to avoid unnecessary medical activities (AOR 1.93, 95% CI 1.26–2.95). More frequently reaching agreement with patients was also associated with finding the campaign very useful (AOR 2.21, 95% CI 1.51–3.24). Among those who did not find the campaign useful, 74% stated that this could partly be due to the campaign’s inability to influence patients’ opinions. 90% of the GPs who were aware of the campaign had implemented one or more recommendations.

**Conclusions:**

Choosing Wisely appears to be considered useful by a substantial proportion of Norwegian GPs. Since the effectiveness of the campaign seems to be linked to the ability to manage gatekeeping, it may be beneficial to bolster the gatekeeping role of GPs and raise public awareness of medical overuse.

**Supplementary Information:**

The online version contains supplementary material available at 10.1186/s12875-025-02928-5.

## Background

It is estimated that one fifth of all medical activity is overuse [1], a problem that has received increasing attention in all clinical fields in recent years [2–5]. The Choosing Wisely initiative has expanded into a global movement involving more than 30 countries [6], aiming to reduce medical overuse and ensure that patients receive the most appropriate and effective treatment. The campaign promotes awareness of unnecessary, low-value medical services, and encourages healthcare professionals and patients to make informed and wise choices regarding patient care [7–9]. Choosing Wisely Norway was launched in 2018 and is directed towards healthcare professionals, patients, and the general population. Most of the Norwegian colleges of health professionals have developed specific Choosing Wisely-recommendations about how to avoid overuse within their field [7].

The issue of overuse is relevant in the context of Norwegian general practice, where GPs have faced an increased workload in recent years. Some GPs report that their working conditions are unsustainable [10]. A heavy workload and work-related stress have also been linked to greater use of unwarranted healthcare services and an increased risk of medical errors [11]. The Norwegian College of General Practice has published ten Choosing Wisely recommendations for commonly used procedures and treatments that are considered to cause more harm than good, and which GPs should avoid in their clinical practice [7].

Campaigns such as Choosing Wisely have the potential to support GPs in their gatekeeping role. It is known that guidelines can have an educational impact on GPs’ practice [12], and that GPs in general have a positive attitude toward guidelines, with high self-reported commitment rates [13–15]. Nevertheless, the extent to which GPs change their practice according to new guidelines and recommendations varies [16, 17].

Several evaluations of Choosing Wisely interventions have been conducted in recent years, mainly in North America. A systematic review of 131 articles concluded that interventions are most effective when they are aimed directly at clinicians and consist of more than one method [18]. Further, a Canadian report from 2022 found that overuse of eight out of 12 specific low-value services was reduced by at least 10% over a five- to six-year period after the introduction of Choosing Wisely [19].

So far, no general assessment of Choosing Wisely initiatives in Norway have been published. It is not known to what extent Norwegian GPs find the campaign useful in curbing medical overuse, nor have possible reasons why Choosing Wisely recommendations may miss their mark been explored, including the possibility that work-related stress might prevent GPs from engaging with Choosing Wisely. The aim of this study was to investigate how Norwegian GPs assessed the usefulness of Choosing Wisely in their clinical practice in general, and to what extent they had changed their practice based on the ten Choosing Wisely recommendations from the Norwegian College of General Practice. Furthermore, we wanted to investigate the characteristics of GPs who perceived the campaign as useful and the reasons why GPs might not find the campaign useful.

## Methods

### Survey design

We conducted a cross-sectional online survey. The questionnaire was developed for this study by the authors and amended with suggestions from three additional researchers (see supplementary File 1 “Questionnaire English version”). A validated short version of the Effort-Reward Imbalance model, to measure work-related stress, was included.

### Setting

Practically all GPs in Norway are members of the Norwegian College of General Practice. All 8,149 members of the college were in August 2021 invited by email to answer an internet-based questionnaire (Questback). Two reminders were sent after three and seven days, respectively, to encourage response. The survey was closed after 15 days. Only practising GPs were included in the analyses.

### The questionnaire

The first part of the questionnaire included seven demographic items: sex, age, time working in general practice, GP-specialist status (trainee or GP specialist), patient list size (number of individuals that each GP is responsible for) and urbanity of office location. Employment status distinguished between GPs who were self-employed and remunerated on a fee-for-service basis, and those who were on a fixed salary (Table 1).

**Table 1:** Baseline characteristics of 900 respondents compared with all GPs in Norway in 2021

	Number of respondents	% (95% CI)	Number of GPs in Norway	% (95% CI)
Sex	900		4968^a^	
Male	429	48% (44,4–50,9)*	2641	53% (51,8–54,4)*
Female	471	52% (49,1–55,6)*	2327	47% (45,5–48,2)*
Age, years	900		4968^a^	
mean	46,9	11,2	48,6	
<30	20	2% (1,3–3,2)	65	1% (1,0–1,6)
30–39	245	27% (24,4–30,2)	1402	28% (27,0–29,5)
40–54	391	44% (40,3–46,8)	2134	43% (41,6–44,3)
55–66	199	22% (19,4–24,9)	1119	23% (21,4–23,7)
>66	43	5% (3,4–6,2)	248	5% (4,38 − 5,60)
Mean years in general practice	14,7		-	
GP specialist status	900		4956^b^	
GP specialist	60	68% (64,6–70,7)	3162	64% (62,5–65,1)
Trainee	29	32% (29,3–35,4)	1794	36% (34,9–37,5)
Organization of practice	900		4989^b^	
Private practice	705	78% (75,6–81,0)*	4239	85% (84,0–86,0)*
Fixed salary	195	22% (19,0–24,4)*	750	15% (14,0–16,0)*
Number of patients listed	900		5167^b^	
Mean	1049	(1027–1070)	1068	
0-1000	445	49% (46,2–52,7)	2403	46% (45,1–47,9)
1001–1500	396	44% (40,8–47,2)	2358	46% (44,3–47,0)
>1500	59	7% (4,9 − 8,2)	406	8% (7,1–8,6)
Population in practice municipality	900		4960^b^	
0–4 999	106	12% (9,7–13,9)	493	10% (9,1–10,8)
5 000–49 999	430	48% (44,5–51,0)	2348	47% (46,0–48,7%
> 50 000	364	41% (37,2–43,7)	2119	43% (41,3–44,1)

^a^Statistics Norway

^b^The Norwegian Directorate of Health

*Significant difference between respondents and GP population

We aimed to explore how the participants typically managed patient requests for unnecessary medical interventions and whether this influenced the perceived usefulness of Choosing Wisely Norway. Two items were scored on two different four-point Likert scales: How frequently the GP had received requests from patients or their relatives that the GP considered unnecessary, ranging from at least daily to less than monthly; and how frequently the GP reached an agreement with the patient or their relatives not to fulfil such a request, ranging from always to rarely/never.

We also wanted to investigate whether work-related stress influenced the GPs’ perception of how useful the campaign was. The Effort Reward Imbalance included nine items (four effort items and five reward items) scored on five-point Likert scales [20]. In the model, we calculated the ratio of the mean score of the effort questions to the mean score of the reward questions. ERI values higher than 1.0 signify a high level of job stress [21].

**Fig. 1:**
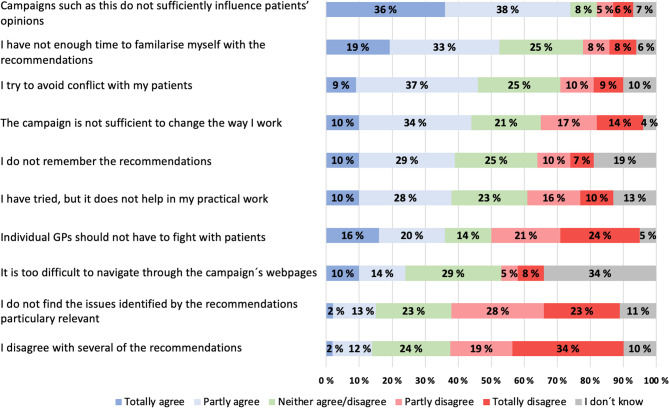
Possible reasons why GPs did not find Choosing Wisely Norway useful in redusing over diagnosis and over treatment. Please indicate how strongly you agree with the following statements of possible reasons why you do not find the campaign useful in reducing overdiagnosis/overtreatment in your clinical practice. (*N* = 137)

The section regarding Choosing Wisely in the questionnaire consisted of 22 items. Among GPs stating that they were familiar with the campaign, we asked to what extent Choosing Wisely Norway had been useful in reducing overdiagnosis and overtreatment in their clinical work in general. The responses were given on a four-point Likert scale, ranging from very useful to not at all useful. Possible reasons for not perceiving the campaign as useful were investigated through ten statements, and responses were given on a five-point scale ranging from 1 = totally agree, to 5 = totally disagree (Fig. 1).

**Fig. 2:**
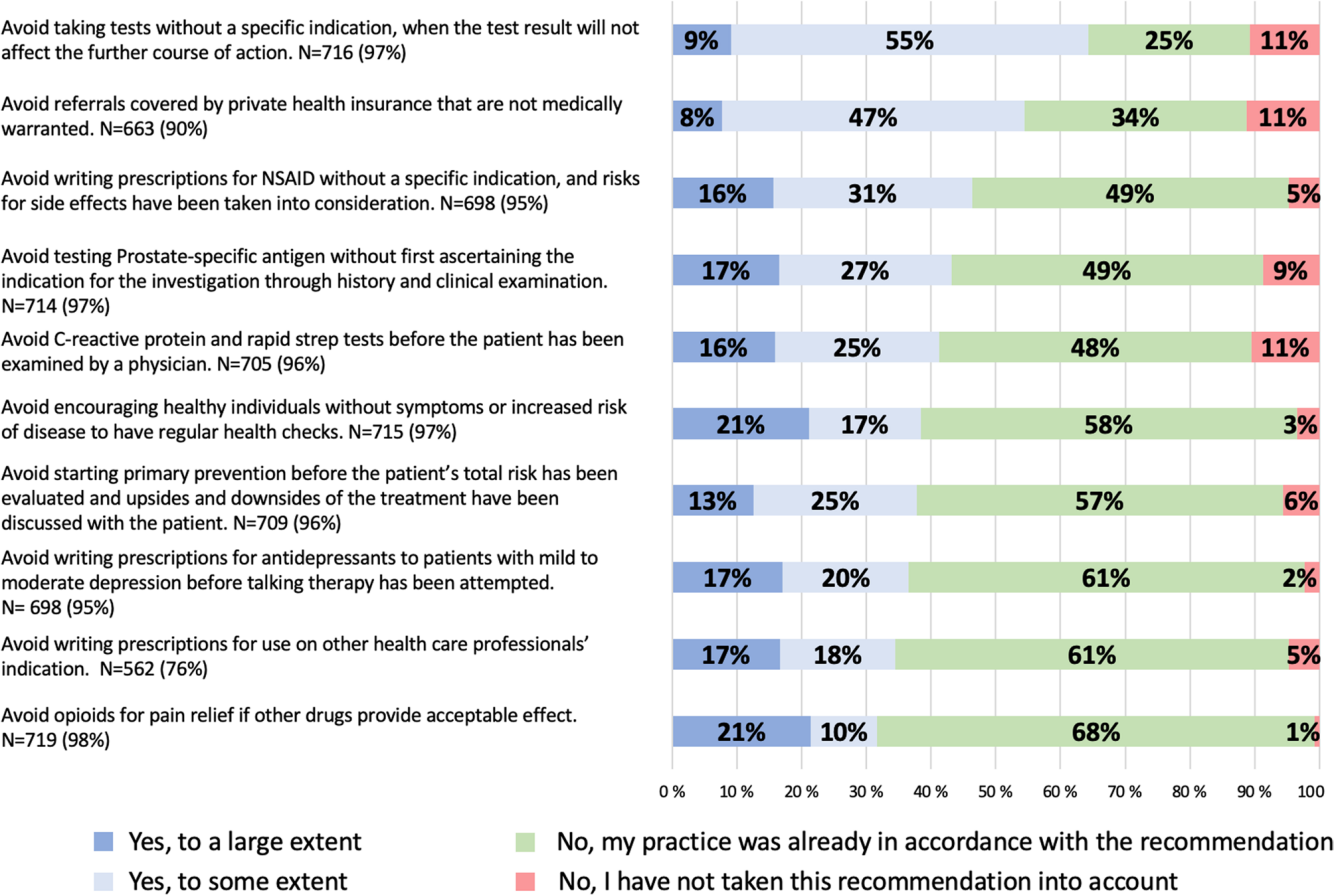
GPs’ self-reported implementation of recommendations from Choosing Wisely Norway. Have you changed your clinical practice according to the following recommendations from the Norwegian Choosing Wisely campaign? The ten specific recommendations with the percentage distribution and the total number of those reporting being aware of each recommendation

We explored to what extent respondents had changed their clinical practice based on ten specific recommendations from Choosing Wisely Norway (Fig. 2).

The questionnaire responses had the following options: (1) yes, to a large extent; (2) yes, to some extent; (3) no, my practice was already in accordance with the recommendation; (4) no, I have not taken this recommendation into account; and (5) I was not aware of this recommendation.

### Statistical analyses

Descriptive statistics with means, proportions, and confidence intervals (CI) were used to describe the sample. CIs were calculated using the following formula: CI= ± (z x SE), where the z-value is 1,96 corresponding to a 95% confidence level, and the standard error (SE) = √(p x (1-p)/n) [22].

Using the ordinal dependent variable *perceived usefulness of Choosing Wisely Norway in reducing overdiagnosis and overtreatment in clinical practice*, we conducted an ordinal regression. We used three ranked categories (not so/not at all useful, somewhat useful, and very useful) in the ordinal regression analyses. Sex, age, list size, experience in general practice and organisation of practice were chosen as covariate variables. In addition, GP-specialist status and the population size of the practice municipality, the frequency of unnecessary requests, frequency of reaching agreement and the Effort-Reward imbalance ratio were included in the initial bivariate ordinal regression analysis. Spearman’s rank correlation was used to check all variables for multicollinearity. The selection of predictors for the final adjusted multiple logistic regression model was conducted using a stepwise forward hierarchical manual method. This process began with a model that included only the five covariate variables. The other independent variables with p-values < 0.05 after the bivariate regression were then added in a hierarchical order of inclusion until the p-value for the regression model exceeded 0.05. We calculated odds ratios (OR) and confidence intervals (CI) and considered *p*-values of < 0.05 to be statistically significant. We used STATA™ statistical software version 17.0 for the regression analysis.

## Results

### Characteristics of the respondents

One thousand one hundred twenty one members of the Norwegian College of General Practice answered the survey. Non-GPs and retired doctors were excluded. The final sample consisted of 900 respondents, comprising 18% of 4,968 practising GPs in Norway. Table 1 presents the demographic characteristics of participating GPs compared with all Norwegian GPs in 2021. Of the respondents, 42% (*n* = 378) had received daily requests from patients for medical services that they considered unnecessary (Table 2).

**Table 2:** GPs’ perceptions of patient requests for unnecessary medical interventions, work-related stress, and choosing wisely Norway in 2021

	Respondents *n*	% (95% CI)
How often do patients request an examination/referral/treatment that you consider unnecessary?	*N* = 900	
> Daily	378	42% (39.0-45.5.)
< Daily and > weekly	419	47% (43.6–50.1.)
< Weekly and > monthly	87	10% (7.9–11.8)
< Monthly	11	1% (0.6–2.2)
Not sure	5	0,5% (0.0–1.0)
Faced with a patient request that you consider unnecessary, how often do you and the patient reach an agreement not to execute the course of action that the patient has requested?	*N* = 900	
Always	20	2% (1.4–3.4)
Often	496	55% (51.8–58.4)
Sometimes	348	39% (35.6–41.9)
Rarely/never	35	4% (2.8–5.3)
Not sure	1	0,1% (0.0-0.3)
Effort-Reward Imbalance Ratio	*N* = 900	
High level of work-related stress (> 1)	362	40% (37,0–43,5)
Low level of work-related stress (< 1)	538	60% (56,4–63,0)
Are you aware of Choosing Wisely Norway?	*N* = 900	
Yes	733	81% (78,9–83,9)
No	147	16% (14,1–18,9)
Not sure	20	2% (1,4 − 3,4)
How useful is Choosing Wisely Norway in reducing overdiagnosis/overtreatment in your clinical work?	*N* = 733	
Very useful	173	24% (20,6–26,7)
Somewhat useful	425	58% (54,2–61,4)
Not so useful	94	13% (10,7–15,6)
Not at all useful	16	2% (1,3–3,5%)
I don’t know	25	3% (2,4–5,1)

57% (*n* = 516) reported that in such consultations they would often or always reach an agreement with the patient not to fulfil the patient’s initial request. 40% of the participants had ERI greater than 1.0, indicating high levels of work-related stress.

### Perceptions of choosing wisely Norway

81% (*n* = 733) respondents were aware of Choosing Wisely Norway. Of these, 24% (*n* = 173) reported that the campaign was very useful in reducing overdiagnosis and overtreatment in their clinical work, and 58% (*n* = 425) reported that it was useful to some extent.

Among those respondents who were aware of the campaign and were familiar with the specific recommendations issued by the Norwegian College of General Practice, 80% (*n* = 520) reported that their practice was already in accordance with one or more of the recommendations before they were published, and 50% (*n* = 329) reported practising in accordance with six or more of the recommendations. 90% (*n* = 585) reported having changed their practice to a large or some extent, based on one or more of the ten Choosing Wisely recommendations. Adopting four or more recommendations was reported by 47% (*n* = 305), and adopting all ten recommendations was reported by 12% (*n* = 78) of respondents.

The highest effect score was for the recommendation *Avoid taking tests unless there is a specific indication*,* and the test result will affect the further course of action* (Fig. 2). 64% (*n* = 461) of participants reported that they had changed their clinical practice to a large or to some extent based on this recommendation, and it was also the recommendation that fewest (25%, *n* = 178) reported that their practice was already in compliance with. In general, fewer GPs reported changing their practice according to those recommendations which a high proportion of GPs had already been practising in accordance with. This is illustrated by the recommendation: *Avoid writing prescriptions for opioids if other drugs give acceptable pain relief.* 68% (*n* = 486) of respondents reported that they were already practising in accordance with this recommendation, whereas 32% (*n* = 228) reported that they had changed their practice based on the recommendation. The proportion of respondents who had not taken the recommendations into account ranged from one to eleven%.

Figure 1 shows possible reasons GPs reported for not finding Choosing Wisely Norway useful in reducing overdiagnosis and overtreatment. Among the 15% (*n* = 111) of respondents who were aware of the campaign, but reported that they did not find it useful, 74% (*n* = 82) totally or partially agreed that this could be due to the campaign’s inability to influence patients’ opinions; while 52% (*n* = 58) believed that lack of time to familiarise themselves with the recommendations, was a contributing factor.

### Ordinal regression analysis

As shown in Table 3, perceiving Choosing Wisely Norway as very useful or somewhat useful in reducing overdiagnosis and overtreatment was significantly associated with more frequently reaching agreement with patients to avoid unnecessary medical activities (adjusted odds ratio (AOR) 1.93, 95% confidence interval (CI) 1.26–2.95). Perceiving the campaign as very useful or somewhat useful also correlated with lower levels of work-related stress (AOR 0.61, 95% CI 0.45–0.83). Perceiving the campaign as very useful correlated with being on a fixed salary (AOR 1.64, 95% CI 1.06–2.57), being female (AOR 1.66, 95% CI 1.15–2.39 or more frequently reaching agreement with patients to avoid unnecessary medical procedures in general (AOR 2.21, 95% CI 1.51–3.24). There was no correlation between the perceived usefulness of the campaign and other demographic variables or how frequently the GPs had perceived requests as unnecessary.

**Table 3:** Ordinal regression of factors associated with perceiving Choosing Wisely Norway as useful (*N* = 707^a^)

		Crude ordinal regression		Adjusted ordinal regression	
		Very/somewhat useful vs. not so/not at all useful	Very useful vs. somewhat/not so/not at all useful	Very/somewhat useful vs. not so/not at all useful	Very useful vs. somewhat/not so/not at all useful
Independent variables		OR (95% CI)	OR (95% CI)	OR (95% CI)	OR (95% CI)
Sex^b^	Male^c^ or Female	1.48 (0.99–2.23)	1.49 (1.05–2.11)*	1.42 (0.92–2.12)	1.66 (1.15–2.39)**
Age^b^	(continuous)	0.97 (0.96–0.99)**	0.99 (0.98–1.01)	0.96 (0.92-1.00)	1.00 (0.96–1.04)
Years working in general practice^b^	(continuous)	0.98 (0.96–0.997)*	1.00 (0.98–1.01)	1.02 (0.97–1.06)	1.00 (0.96–1.04)
Organization of practice^b^	Private practice^c^ or fixed salary	1.36 (0.80–2.31)	1.57 (1.05–2.33)*	0.92 (0.52–1.65)	1.64 (1.06–2.57)*
Number of patients listed^b^	(continuous)	1.00 (1.00–1.00)*	1.00 (1.00–1.00)	1.00 (1.00–1.00)	1.00 (1.00–1.00)
GP specialist status	GP specialist^c^ or trainee	1.30 (0.82–2.04)	1.05 (0.73–1.51)	^d^	
Population in practice municipality	(ordinal)^e^	0.96 (0.79–1.17)	1.12 (0.95-1-31)	^d^	
How often do patients request an examination, referral or treatment, that you consider as unnecessary?	(ordinal)^f^	0.87 (0.64–1.19)	1.30 (1.00-1.70)	^d^	
If you get a patient request like that, how often do you and the patients reach an agreement to not execute the course of action asked?	(ordinal)^g^	1.81 (1.21–2.73)**	2.07 (1.43-3.00)***	1.93 (1.26–2.95)**	2.21 (1.51–3.24)***
Effort-Reward Imbalance ratio	(continuous)	0.61 (0.46–0.81)**	0.87 (0.65–1.17)	0.61 (0.45–0.83)**	0.92 (0.68–1.25)

Statistically significant odds ratios for the adjusted ordinal regression are highlighted in bold

^a^Participants who reported being aware of Choosing Wisely Norway. 26 missing

^b^Demographic variables included as covariate variables

^c^Reference for dummy variables

^d^Not included in final model because of *p* > 0.05 in bivariate ordinal regressions

^e^Categories ranked: less than 10 000, between 10 000 and 30 000, between 30 000 and 100 000, more than 100 000

^f^Categories ranked: less than weekly, less than daily but at least weekly, at least daily

^g^Categories ranked: Rarely/never, Sometimes, Often, Always

*p < 0.05

**p < 0.01

*** *p* < 0.001

## Discussion

### Summary

Most of the respondents were aware of Choosing Wisely Norway. Of these, more than eight out of ten found the campaign very or somewhat useful in reducing overdiagnosis or overtreatment in their clinical work. Even though many GPs had already been practising in accordance with the Choosing Wisely recommendations, a large majority reported that they had implemented one or more of the recommendations in their practice. GPs who reported being on a fixed salary, were female or more frequently came to an agreement with patients not to conduct medical activities that the GP considered unnecessary, perceived the campaign most useful. In contrast, higher levels of work-related stress and less frequently reaching agreement with patients was associated with perceiving the campaign as less useful. Among respondents who did not find the campaign useful, two out of three stated that this could partly be due to the campaign’s inability to influence patients’ opinions.

### Strengths and limitations

To our knowledge, this is the first study to evaluate how GPs in Norway perceive a campaign aimed at reducing medical overuse in clinical practice. Almost all GPs in Norway are members of the Norwegian College of General Practice and therefore received an invitation to participate in the survey.

The response rate for surveys among doctors is often low [23, 24], as was the case for our study (18%). An extended period of data collection might have obtained more responses. However, our previous experiences regarding the challenges of obtaining a high yield in such surveys lead us to believe that this would have had limited effect.

Although the responding GPs were similar to GPs in Norway regarding age and some other background variables, the higher proportion of females and GPs with a fixed salary indicates a possible sampling bias.

We suspect that the results are not fully representative of the entire GP population. Unfortunately, we could not investigate the reasons for GPs choosing not to participate. The low response rate might also indicate that the campaign does not appeal to all GPs in Norway. GPs with greater knowledge of medical overuse or interest in the topic, or with a positive attitude to initiatives such as Choosing Wisely, may have been more likely to participate than others [25]. It is also possible that respondents exaggerated their positive attitude and perceptions of the campaign. In general, self-reported opinions and behaviour may differ from actual performance [26, 27]. Thus, the proportion of Norwegian GPs who are aware of Choosing Wisely may be lower than in our sample, and the usefulness of the campaign and specific recommendations from Choosing Wisely may also be lower than indicated by our findings.

While the results must be interpreted in the context of the Norwegian healthcare system, where GPs act as gatekeepers to secondary healthcare, the perceptions of clinicians on Choosing Wisely Norway may nevertheless be relevant for other countries with a similar, universal healthcare system facing the challenges of medical overuse.

### Comparison with existing literature

Few studies have investigated GPs’ awareness of Choosing Wisely. In a survey from 2022, three quarters of primary care physicians from six high-income countries were aware of Choosing Wisely recommendations [19]. An American survey revealed that nearly half of all primary care physicians were aware of the campaign two years after it had been launched [28]. However, general awareness of campaigns or guidelines is not the same as being familiar with their content [24, 29, 30]. Our results indicate that the campaign is helpful for a significant proportion of Norwegian GPs. This is important considering the challenges of medical overuse in healthcare services. Choosing Wisely recommendations may act as an eye opener for GPs, and serve as a reminder for those who have not practised in accordance with their knowledge [31]. A survey published in 2020 found that Swiss GPs perceived many Choosing Wisely recommendations to be highly relevant in their clinical practice, regardless of their prior knowledge of the recommendations [32]. Furthermore, the recommendations may also serve as a tool for improved communication with patients, as reported from Canada [33]. It is known that GPs sometimes refer to third parties such as guidelines when turning down requests from patients [34]. An Australian study from 2023 also suggested that clear guidelines for proper use of radiology could be both educational and reassuring for anxious patients [35].

We found an association between the perceived usefulness of Choosing Wisely and how frequently the participants reached an agreement with their patients not to accommodate requests for health services the GP did not consider to be medically warranted. We suggest that this is likely to hold true for Norwegian GPs in general and not just for our sample. According to implementation research, guidelines are implemented in several stages [31]: (1) knowledge of new guidelines; (2) awareness of the need for change; (3) intention to change; and (4) implementation. This is also in line with a study among Swiss GPs, who reported adherence of between 80% and 99% to the top ten Choosing Wisely recommendations they agreed with [27]. Furthermore, doctors who perceive medical overuse as a problem in their practice are more likely to report being familiar with Choosing Wisely [24]. GPs who are already committed to the role as gatekeeper may be more receptive to campaigns addressing this issue than colleagues with less awareness or less willingness to act as gatekeepers. Understanding and maintaining gatekeeping is complex and requires a high degree of medical professionalism [36]. Hence, there seems to be a need to bolster the gatekeeping role of GPs.

In this study, higher level of work-related stress was associated with limited usefulness of the campaign. In the years preceding our survey, there has been an observed increase in self-reported work-related stress among Norwegian GPs, from 10 to 40% through a ten-year period [37], and this has been linked to an increase in workload [38, 39]. Burnout among Danish GPs has also been shown to correlate with lower threshold for referring patients to specialist care [40]. Physician stress is likely to impair the capacity to provide high-quality healthcare for patients, and it may be less burdensome to comply with patients’ requests rather than attempting to persuade patients to follow other courses of action that align more closely with professional guidelines [11, 41]. The implementation of guidelines requires communication skills and time for reflection, which can be challenging under high work pressure.

The proportion of female respondents was significantly higher than in the total Norwegian GP population, which is similar to the distribution of respondents in an American survey from 2016 exploring doctors’ knowledge of, and feelings towards, Choosing Wisely [28]. Furthermore, in our survey, being female was significantly associated with finding the campaign very useful in reducing overdiagnosis or overtreatment. Previous research has shown that female doctors have longer consultations and are more empathic and patient-centred in their communication compared with their male colleagues [42, 43]. Such qualities can be helpful in reaching agreements with patients. Previous qualitative studies from Norwegian general practice have suggested that improving communication skills in general and especially using patient-centred approaches to build trust and reach common ground, can be of use for negotiating potential conflicts with patients [44, 45].

Almost two thirds of the respondents who did not find the campaign useful, totally or partially agreed that this could partly be due to the campaign’s inability to influence patients’ opinions. Patients’ expectations are a known barrier to evidence-based practice [46] and a driver of medical overuse for GPs [25]. Referrals based primarily on demands from patients are common in Norway [47], and one survey found that 43% of GPs considered patient preferences an important reason for referral [48]. GPs are uncomfortable with turning down patients’ requests and fear the negative consequences of such rejections [44, 49]. Thus, Embrett and Randall have argued that recommendations regarding medical overuse often fail to consider the various pressures that contribute to unnecessary care. They suggest that there is a need to improve the communication skills of doctors and to increase awareness about medical overuse not only among healthcare professionals, but among the general public as well [50].

## Conclusions

Even though our survey had a low response rate and bias may be involved, our findings indicate that a considerable proportion of GPs in Norway find Choosing Wisely to be useful in reducing medical overuse. This is particularly noticeable among GPs who frequently reach agreement with patients to avoid unwarranted services. It is therefore possible that the campaign helps reinforce existing practices among GPs who already are committed to minimising overuse. Additionally, the low response rate combined with the fact that GPs who reported high levels of work-related stress also found the Choosing Wisely to be less helpful, may indicate that not all GPs in Norway find it important to address medical overuse and gatekeeping. Given that the effectiveness of the campaign may be linked to the ability to manage this role, it appears important to consider ways to strengthen GPs’ capacity as gatekeepers and reduce work-related stress. Moreover, it may be beneficial to increase awareness of medical overuse among the general public. Future research could explore how patients perceive gatekeeping in general practice and directly measure the impact of Choosing Wisely in Norway on patient treatment.

## Supplementary Information

Supplementary Material 1.

## Data Availability

The datasets used and analysed during the current study are available from the corresponding author on reasonable request.

## Abbreviations

GP: General practitioner
AOR: Adjusted odds ratio
CI: 95% confidence interval
