# Supplementary material for: Perceptions of the usefulness of Choosing Wisely among general practitioners in Norway: a nationwide survey
Source: BMC Prim Care. 2025 Aug 2;26:240. doi: 10.1186/s12875-025-02928-5 (PMC12318425; doi:10.1186/s12875-025-02928-5)
Supplement: Supplementary file 1 — Supplementary Material 1. [file 12875_2025_2928_MOESM1_ESM.docx]

**Questionnaire**

The following is an English translation of the questionnaire that was presented to the participating GPs in Norwegian.

1. Sex

Female

Male

1. What is your year of birth?
2. How many years have you worked in general practice?
3. Are you a GP specialist?

Yes

No

1. What is your primary workplace?

Doctors´ surgery

Out of hours clinic

Nursing home

Specialist health service

Academic position

Municipal administration

Pensioner

Other

1. Are you currently working as a GP?

Yes

No

1. How many residents live in the municipality where you work as a GP?

0 – 1 999

2 000 – 4 999

5 000 – 9 999

10 000 – 19 999

20 000 – 29 999

30 000 – 49 999

50 000 – 99 999

>100 000

8. How are your practice organized?

Self-employed

Salaried

Combination of self-employed and salaried

9. How many days a week do you see patients?

10. Approximately how many patients do you have on your GP list?

11. How often do patients request an examination/referral/treatment that you consider unnecessary?

At least daily

At least weekly

At least monthly

Less frequently than monthly

I am not sure/ Not relevant

12.  Faced with a patient request that you consider unnecessary, how often do you and the patient reach an agreement not to execute the course of action that the patient has requested?

Always

Often

Sometimes

Rarely/never

I am not sure/ Not relevant

13. Are you aware of Choosing Wisely Norway?

Yes

No

I am not sure/ It is not relevant

*If you answered* ***No*** *to the last question, skip to question 18.*

14. Indicate whether you have heard about the Choosing Wisely campaign through the following sources:

Yes No

a. International journal

b. Norwegian journal

c. Courses/professional meetings

d Social media

e. Campaign materials

f. The campaign website

g. Colleagues

h. Norwegian Electronic Medical Handbook

i. Other sources

15. How useful is Choosing Wisely Norway in reducing overdiagnosis/overtreatment in your clinical work?

Very useful

Somewhat useful

Not so useful

Not at all useful

I am not sure/ It is not relevant

16. *If you answered* ***Not so useful/ Not at all useful*** *to the previous question, please indicate how much you agree with the following statements about possible reasons why you do not find the campaign useful in reducing overdiagnosis/overtreatment in your clinical work. If you answered* ***Very useful/ Somewhat useful/ I am not sure/ It is not relevant:*** *skip to question 17.*

|  | Totally agree | Partly agree | Neither agree nor disagree | Partly disagree | Totally disagree | I don’t know |
| --- | --- | --- | --- | --- | --- | --- |
| 1. I do not find the issues that are addressed by the recommendations particularly relevant |  |  |  |  |  |  |
| 1. I disagree with several of the recommendations |  |  |  |  |  |  |
| 1. The campaign is not sufficient to change the way I work |  |  |  |  |  |  |
| 1. It is too difficult to navigate through the campaign webpage |  |  |  |  |  |  |
| 1. I do not have enough time to familiarize myself with the recommendations |  |  |  |  |  |  |
| 1. I have tried, but it does not help in my practical work |  |  |  |  |  |  |
| 1. I do not remember the recommendations |  |  |  |  |  |  |
| 1. Campaigns such as this do not sufficiently influence patients´ opinions |  |  |  |  |  |  |
| 1. Individual GPs should not have to fight with patients |  |  |  |  |  |  |

1. Can you think of any other reasons why you do not find the campaign useful?

17. Have the following recommendations from the Norwegian Choosing Wisely campaign led you to change your practice in accordance with the recommendations?

|  | Yes, largely | Yes, to some extent | No, my practice was already in accordance with the recommendation | No, I have not taken this recommendation into account | I have not heard of this recommendation before | I don’t know/ It is not relevant |
| --- | --- | --- | --- | --- | --- | --- |
| - 1. Avoid referrals covered by private health insurance that are not medically warranted. |  |  |  |  |  |  |
| - 1. Avoid writing prescriptions for use on other health care professionals´ indication. |  |  |  |  |  |  |
| - 1. Avoid C-reactive protein and rapid strep test before the patient has been examined by a physician. |  |  |  |  |  |  |
| - 1. Avoid writing prescriptions for antidepressants to patients with mild to moderate depression before talking therapy has been attempted. |  |  |  |  |  |  |
| - 1. Avoid opioids for pain relief if other drugs provide acceptable effect. |  |  |  |  |  |  |
| - 1. Avoid writing prescriptions for NSAID without a specific indication, and risks for side effects have been taken into consideration. |  |  |  |  |  |  |
| - 1. Avoid testing Prostate-specific antigen without first ascertaining the indication for the investigation through history and clinical examination. |  |  |  |  |  |  |
| - 1. Avoid starting primary prevention before the patient´s total risk has been evaluated and upsides and downsides of the treatment have been discussed with the patient. |  |  |  |  |  |  |
| - 1. Avoid encouraging healthy individuals without symptoms or increased risk of disease to have regular health checks. |  |  |  |  |  |  |
| - 1. Avoid taking tests without a specific indication, and the test result will not affect the further course of action. |  |  |  |  |  |  |

1. Consider the following statements about how you feel at your job:

|  | Disagree | Agree and I am not at all distressed | Agree and I am somewhat distressed | Agree and I am distressed | Agree and I am very distressed |
| --- | --- | --- | --- | --- | --- |
| I have constant time pressure due to a heavy workload. |  |  |  |  |  |
| I have a lot of responsibility in my job. |  |  |  |  |  |
| I have many interruptions and disturbances while performing my job. |  |  |  |  |  |
| Over the past few years, my job has become more and more demanding |  |  |  |  |  |
| My job promotion prospects are poor. |  |  |  |  |  |
| I have experienced or I expect to experience an undesirable change in my work situation. |  |  |  |  |  |
| My job security is poor. |  |  |  |  |  |
|  | Agree | Disagree and I am not at all distressed | Disagree and I am somewhat distressed | Disagree and I am distressed | Disagree and I am very distressed |
| Considering all my efforts and achievements, I receive the respect and prestige I deserve at work. |  |  |  |  |  |
| Considering all my efforts and achievements, my salary / income is adequate. |  |  |  |  |  |
